# Synergistic Effect of Cellulose Nanofiber and Nanoclay as Distributed Phase in a Polypropylene Based Nanocomposite System

**DOI:** 10.3390/polym12102399

**Published:** 2020-10-18

**Authors:** Bich Nam Jung, Hyun Wook Jung, DongHo Kang, Gi Hong Kim, Jin Kie Shim

**Affiliations:** 1 Korea Packaging Center, Korea Institute of Industrial Technology, Bucheon 14449, Korea; jbn5666@kitech.re.kr (B.N.J.); kangppp@kitech.re.kr (D.K.); kakamate@kitech.re.kr (G.H.K.); 2 Department of Chemical and Biological Engineering, Korea University, Seoul 02841, Korea; hwjung@grtrkr.korea.ac.kr

**Keywords:** nanocomposites, hybrid, polymer-matrix composites (PCM), physical properties

## Abstract

Since the plastic-based multilayer films applied to food packaging are not recyclable, it is necessary to develop easily recyclable single materials. Herein, polypropylene (PP)-based cellulose nanofiber (CNF)/nanoclay nanocomposites were prepared by melt-mixing using a fixed CNF content of 1 wt %, while the nanoclay content varied from 1 to 5 wt %. The optimum nanoclay content in the PP matrix was found to be 3 wt % (PCN3), while they exhibited synergistic effects as a nucleating agent. PCN3 exhibited the best mechanical properties, and the tensile and flexural moduli were improved by 51% and 26%, respectively, compared to PP. In addition, the oxygen permeability was reduced by 28%, while maintaining the excellent water vapor permeability of PP. The improvement in the mechanical and barrier properties of PP through the production of PP/CNF/nanoclay hybrid nanocomposites suggested their possible application in the field of food packaging.

## 1. Introduction

Various plastics have been used to protect foods from external shocks and environmental factors, such as oxygen and moisture, for increasing their shelf life. Typical examples of the most commonly used plastics include polypropylene (PP), polyethylene (PE), polyamide (PA), polystyrene (PS), polyethylene terephthalate (PET), ethylene vinyl alcohol (EVOH), etc. Plastics are also used to form multilayer structures that lead to materials with improved features owing to the complementation of their own properties [1]. In recent decades, the excessive use of petroleum-based polymers, due to their excellent mechanical and barrier properties and low cost, has caused significant serious problems in terms of waste generation [2,3]. In addition, these multilayered plastics cannot be recycled because two or more plastics coexist. Therefore, there is an imperative need to enhance the performance of a single material as a composite.

PP and PE are widely used polymers with various advantages including low cost, good mechanical and moisture barrier properties, and high recyclability. Among them, PP has the advantage of being used as a packaging material because it has superior transparency and heat resistance compared to PE. However, PP is a hydrophobic polymer with poor oxygen barrier properties and low affinity with hydrophilic fillers. Cellulose nanofibers (CNF) and nano-sized clays are well known for their hydrophilic fillers that improve polymers’ mechanical strength and oxygen barrier properties [4,5,6,7,8]. CNFs are produced by a variety of materials, such as bleached wood pulp and cotton linters, and exhibit a great performance even with low composite content [9]. Nanoclays such as montmorillonite (MMT) consist of several stacked layers with a thickness of around 1 nm, where alumina is located in the center as an octahedral sheet sandwiched between two silica tetrahedral sheets [10]. Thus, the dispersion of impermeable CNF and nanoclay in a polymer matrix to form tortuous paths results in an upgrade of the barrier property due to the delayed passage of permeate gas. Due to these characteristics, studies on nanoclay/natural-fiber-filled polymers used for food packaging have been previously reported [11,12]. In addition, it has been reported from previous studies that CNF and nanoclay increase the crystallinity of the polymer by acting as a nucleating agent in the polymer [13,14].

Several studies on the preparation of PP-based composites using cellulose filler and nanoclays, such as bamboo fiber [15], wheat straw fiber [16], wood flour [17], and microcrystalline cellulose (MCC), have already been reported [18,19]. In addition, CNFs are frequently used to form nanocomposites with nanoclay in poly(lactic acid) (PLA) [20,21,22,23] and paper matrix [4], leading to hydrophilic polymers. Although there are several examples of hydrophilic polymers, studies on the CNF and nanoclay dispersion in hydrophobic polymers, such as PP have not been performed to date. It is still challenging and difficult to uniformly disperse CNF and nanoclay in the PP matrix, which is a hydrophobic polymer. Many attempts have focused on the improvement in the compatibility of PP and hydrophilic fillers, while most of these methods use maleic anhydride grafted PP (PP-g-MA or MAPP) as a compatibilizer. However, although the use of compatibilizers assists the filler dispersion in the polymer matrix, maleic anhydride (MA) plasticizes the polymer chains, thus impairing the mechanical and barrier properties [24]. The plasma treatment of the polymer has recently been proposed as an alternative method to improve the compatibility and maintain the initial advanced properties [25]. In addition, this process is carried out under dry conditions using a solvent-free gas, which can overcome the disadvantages of the conventional wet chemical methods [26,27].

To the best of our knowledge, there are no studies on hybrid composites made from nano-sized cellulose fibers and clays in PP matrices. In this study, PP/CNF/nanoclay hybrid nanocomposites were prepared by melt mixing, a process that is directly applicable to industries and preserves the benefits of nanoclay and CNF in nanocomposites. At a fixed CNF amount of 1 wt %, varying nanoclay amounts were added to evaluate their effect on the dispersion state of the CNF in the PP matrix and to determine the optimal nanoclay weight. To that end, the fabricated PP/CNF/nanoclay hybrid nanocomposites were analyzed for their morphological, structural, thermal, mechanical, and barrier properties.

## 2. Materials and Methods

### 2.1. Materials

Freeze-dried CNF, with an average width of 32 nm and several millimeter length, was obtained from Cellulose Lab (Canada). Polypropylene (PP), T3410, was from LG Chem. (Korea). The nanoclay (cloisite 20A) was used as purchased from BYK Additives and instruments (USA). Dimethyl dehydrogenated tallow amine, where most of the double bonds were hydrogenated, was the quaternary ammonium source used as an organic modifier for the nanoclay. A layer of nanoclay has a thickness of about 1 nm, the width of a sheet is around 500 nm, and the dried particle size is 10 μm. Sodium hydroxide (NaOH) was supplied by Sigma Aldrich (USA) has a purity of over 98%.

### 2.2. Preparation of PP/CNF and PP/CNF/Nanoclay Nanocomposites

PP was pulverized by a cryo-grinding method to produce the micro-powder particles. Subsequently, the produced micro-powder PP was plasma-treated. Nitrogen (N_2_) gas was injected into the chamber, and a radio-frequency (RF) plasma power of 50 W was performed to a 13.56 MHz RF generator (Model: FEMTO Version E, Diener electronics GmbH & Co KG, Germany). The micro-powder PP was treated with plasma for an exposure time of 10 min. Furthermore, the micro-powder PP was scattered on a petri dish as a mono layer to expose the plasma. A CNF suspension was prepared by adding 0.4 g CNF and 1 mg NaOH to 25 mL distilled water. The CNF/nanoclay suspensions were prepared by adding different amounts of nanoclay (0.4, 1.2, 2.0 g) to the CNF suspension, followed by homogenization at 6000 rpm for 0.5 h using a disperser (Model: T10 basic, IKA, Germany).

All the PP/CNF and PP/CNF/nanoclay nanocomposites were manufactured using an internal mixer (Model: W50 Plastograph^®^, Brabender GmbH & Co KG, Germany) equipped with a twin-screw with a temperature of 190 °C and 80 rpm rotation speed. The modified micro-powder PP and the suspension were first pre-mixed for 1 min, and then melt-mixed for 5 min in the internal mixer. The liquid contained in the suspensions was evaporated during the melt-mixing process. The PP/CNF and PP/CNF/nanoclay nanocomposites that were generated in this study are listed in Table 1.

### 2.3. Characterization

#### 2.3.1. Sample Preparation

**Figure 1:**
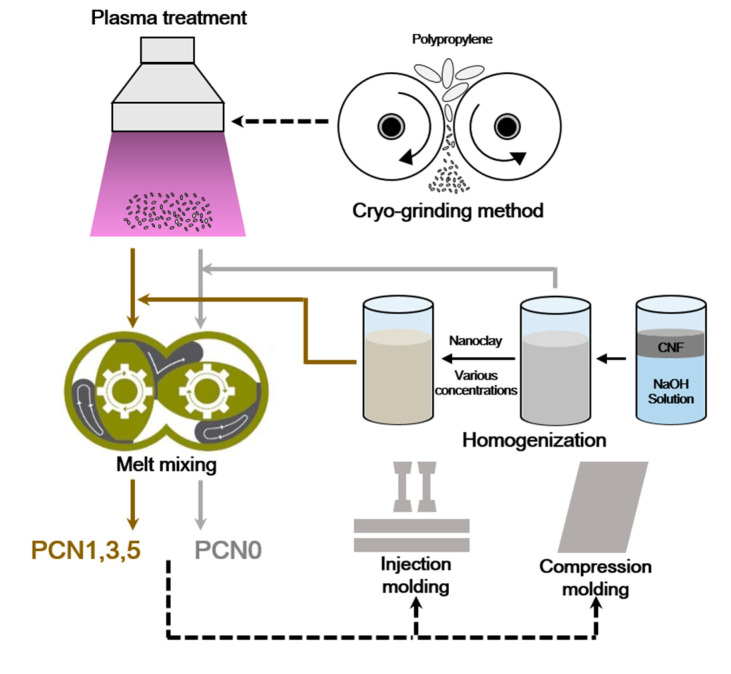
Schematic illustration of PP/CNF and PP/CNF/nanoclay nanocomposites preparation.

The bulk-shaped nanocomposites were ground and dried at 110 °C for 12 h to sufficiently remove the remaining moisture, and the obtained nanocomposite granules were molded by compression to a film type to measure their structural, thermal, and barrier features, as well as their morphological and mechanical properties. The compression molding was performed using a hot press system (Model: QM900A, Qmesys, Korea) operating at 190 °C through a 4 min pre-heating, 1 min pressing, and 4 min cooling step process. The nanocomposite granules were also molded by injection at 190 °C into test specimens with a dumbbell shape of 50 mm length, 4 mm width, and 2 mm thickness and a bar shape of 100 mm length, 10 mm width, and 4 mm thickness, using a micro injection molding machine (Model: Xplore Micro 10 cc Injection Molding Machine, Xplore Instruments, The Netherlands). Schematic illustration of PP/CNF and PP/CNF/nanoclay nanocomposites are described in Figure 1.

#### 2.3.2. Plasma Treatment Efficiency Analysis

Water contact angle analysis was carried out using a contact angle analyzer (Model: SmartDrop, FEMTOFAB, Korea) at room temperature and relative humidity of 30%. Measurement was performed by dropping 3 μL of deionized water at least five times on the surface of PP.

X-ray photoelectron spectroscopy (XPS, Model: K-alpha, Thermo scientific, UK) was used to measure the elemental composition of PP and plasma-treated PP.

#### 2.3.3. Morphological Analysis

The morphology of the PP/CNF and PP/CNF/nanoclay nanocomposites was observed with field emission scanning electron microscopy (FE-SEM, Model: SU8020, Hitachi, Japan). The dumbbell-shaped samples were cryo-fractured using liquid N_2_ to prevent deformation during fracture. All the samples were coated with a Pt/Pd alloy using an ion sputter (Model: E-1045, Hitachi, Japan). The interlayer nanoclay structure of the nanocomposites was analyzed by transmission electron microscopy (TEM, Model: Tecnai F20 G2, FEI, USA). The TEM samples (~50 nm thick) were prepared from the nanocomposite that was extracted from the granule using the cryo-microtome (Model: LEICA ULTRACUT UC7, Leica, Germany) equipped with a diamond knife at −80 °C.

#### 2.3.4. X-ray Diffraction Analysis

The nanoclay dispersion and structural analyses of the film type nanocomposites were performed by X-ray diffraction (XRD, Model: EMPYREAN, PANalytical, Germany) with Cu Kα radiation and a wavelength of 1.5406 Å. The diffraction spectra were obtained at a 2*θ* range of 2°–30°.

#### 2.3.5. Thermal Analysis

Thermogravimetric analysis (TGA, Model: Q500, TA instruments, USA) was used to assess the thermal stability and the residual amounts of nanoclay in all nanocomposite films. The tests were performed under N_2_ atmosphere at temperatures of up to 600 °C with a 10 °C/min heating rate. At least three measurements were taken for each sample.

#### 2.3.6. Mechanical Analysis

The mechanical properties, such as tensile stress, elongation at break, flexural strength, and modulus, were measured with a universal testing machine (UTM, Model: INSTRON 3367, INSTRON, USA) equipped with a 30 kN cell force in accordance with ASTM D638 and ASTM D790. The tensile and flexural test speeds were measured at 100 and 10 mm/min, respectively. In addition, the gauge length during the tensile test was fixed at 19 mm. Tensile and flexural tests were measured with dumbbell and bar-shaped specimens, respectively. At least seven samples were tested for each nanocomposite sample, and five values were applied, except for the maximum and minimum values.

#### 2.3.7. Barrier Analysis

The oxygen permeability measurements of PP and the PP/CNF and PP/CNF/nanoclay nanocomposite films were recorded using an oxygen transmission rate analyzer (Model: 702, MOCON, USA) that complied with ASTM 3985. The permeation tests were performed at 23 °C and 0% relative humidity under 100% O_2_ atmosphere. The water vapor permeability of all the samples was measured by a water vapor transmission rate analyzer (Model: W700, MOCON, USA), according to ASTM F372, while the permeation test was performed at 37.8 °C and 100% relative humidity. The oxygen and water vapor transmission rates were recorded over a period of 24–48 h until a steady state was achieved. At least three measurements were taken for each sample.

#### 2.3.8. Statistical Analysis

Multiple comparisons of each experimental value set were analyzed by means of one-way ANOVA and Tukey’s honestly significant differences test (*α* = 0.05) from IBM SPSS Statistics version 23 (IBM SPSS).

## 3. Results and Discussion

### 3.1. Plasma Treatment Efficiency Properties

**Figure 2:**
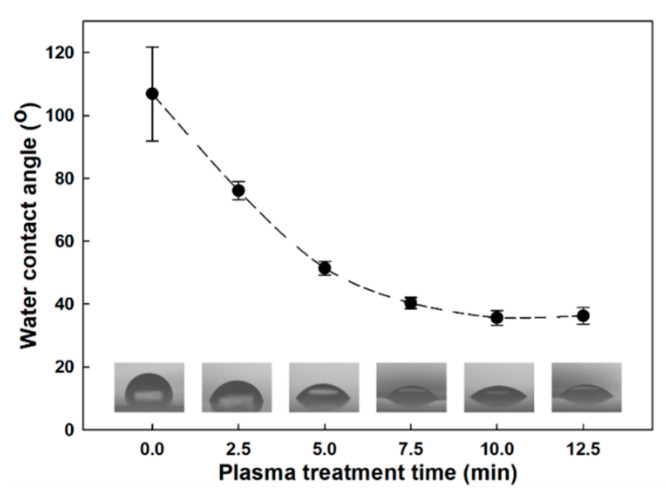
Water contact angle as a function of plasma treatment time for PP surface.

In this experiment, a plasma treatment method was used to improve the affinity between PP and hydrophilic fillers, CNF and nanoclay. The water contact angle of the PP surface was measured while increasing the plasma treatment time, and the optimal plasma treatment time was applied as the experimental condition based on the results. In Figure 2, PP without plasma treatment showed a water contact angle of about 107 ° and exhibited hydrophobic properties. As the plasma treatment time increased up to 10 min, the water contact angle of the PP surface tended to decrease continuously, and it was confirmed that it decreased to 36 °. However, when the PP surface was plasma-treated for more than 10 min, the water contact angle did not decrease any more, indicating that there was saturation of the plasma effect on the PP surface. Therefore, 10 min was selected as the plasma treatment time condition in this experiment.

**Figure 3:**
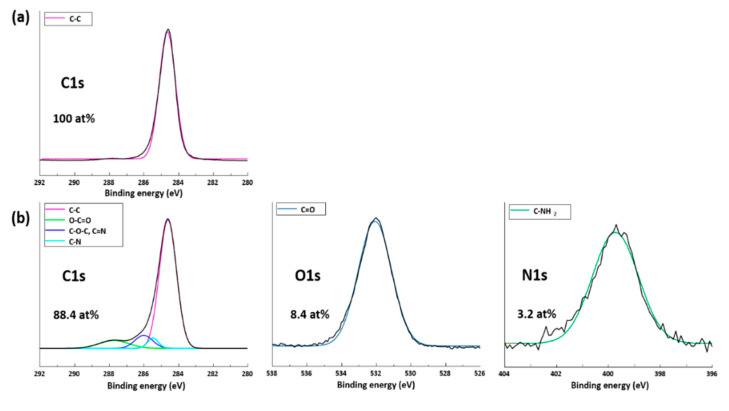
C1s, O1s, and N1s regions of XPS spectra of (**a**) PP and (**b**) plasma-treated PP.

Figure 3 shows XPS spectra demonstrating the effect of surface modification by plasma treatment. The PP treated with plasma for 10 min contained four peaks at 287.4 (O-C=O), 286.5 (C-O-C, C=N), 286.4 (C-N), and 284.6 (C-C) eV in C1s spectrum, and one peak each at 533 (C=O), ~400 eV (C-NH_2_) in the O1s and N1s spectrum (Figure 3b). It was confirmed that the atomic concentrations of the O1s and N1s peaks of the plasma-treated PP increased from 0 to 8.8% and 3.2%, respectively, compared to PP, and the affinity between the fillers and the PP matrix was expected to be improved due to the effect of the plasma treatment.

### 3.2. Morphological Properties

**Figure 4:**
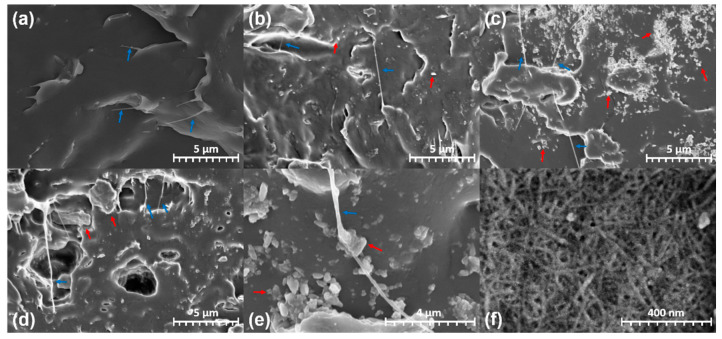
SEM micrographs of CNF and fractured surface images of PP/CNF and PP/CNF/nanoclay nanocomposites including CNF (blue arrow) and nanoclay (red arrow): (**a**) PCN0, (**b**) PCN1, (**c**) PCN3, (**d**) PCN5, (**e**) nanoclays attached to the CNF surface in the PP matrix (PCN3), and (**f**) CNF.

Figure 4 illustrates the SEM images of the fractured surfaces of the PP/CNF and PP/CNF/nanoclay nanocomposites with different magnifications. As observed in Figure 4a, PCN0 was dispersed in individual fibrous forms in the PP matrix. However, the increase in the nanoclay content altered the morphology. More specifically, in PCN1 (Figure 4b), the nanoclay particles were uniformly distributed around the CNF on the fibrous form, while the nanoclay was slightly aggregated when its content was higher than 3 wt % (Figure 4c). The maximum nanoclay agglomeration was observed when the nanoclay content was 5 wt % (Figure 4d). In addition, the nanoclay attachments to the surface of CNF in PCN3 and PCN5 could be clearly observed (Figure 4e). This interaction between the CNF and the nanoclay is based on organic–inorganic hydrogen bonds and can affect the physico-mechanical properties of CNF [28,29,30]. In addition, the nanoclay–CNF interaction suggested that the CNF may induce nanoclay agglomeration with increasing nanoclay content in the nanocomposites.

**Figure 5:**
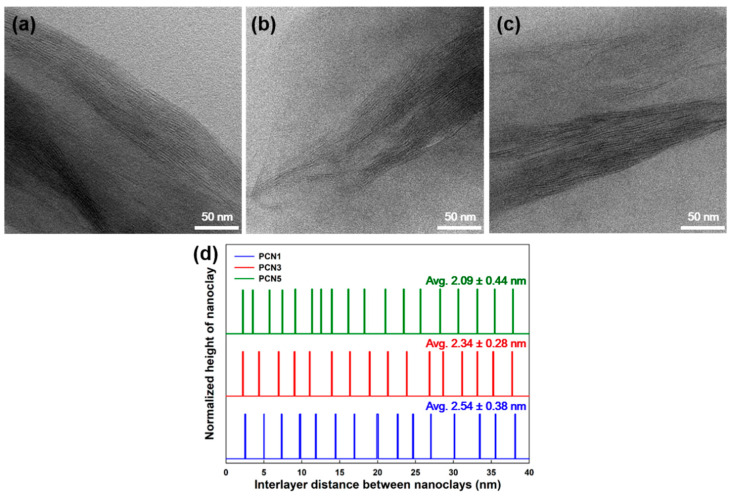
TEM micrographs of cross-section images of the (**a**) PCN1, (**b**) PCN3, and (**c**) PCN5 nanocomposites, and (**d**) interlayer distance of the intercalated nanoclay.

Figure 5 displays the results of TEM analysis of the PP/CNF/nanoclay nanocomposites. Overall, the CNF did not appear to be distinct from the PP matrix phase. In Figure 5a–c, the nanoclays were partially intercalated and exfoliated, while the interlayer distance was measured only in the intercalated part, except for the part where the nanoclay was exfoliated (Figure 5d). In particular, in the PP/CNF/nanoclay nanocomposites, the increase in the nanoclay content from 1 to 5 wt % led to agglomeration of the nanoclay due to the insufficient penetration of the PP chains, which in turn reduced the interlayer distance of the nanoclay from 2.54 to 2.09 nm. Similar results were observed in nanoclay-based nanocomposites developed by Arora et al. [31] and Decker et al. [32].

### 3.3. Nanoclay Dispersion Properties

**Figure 6:**
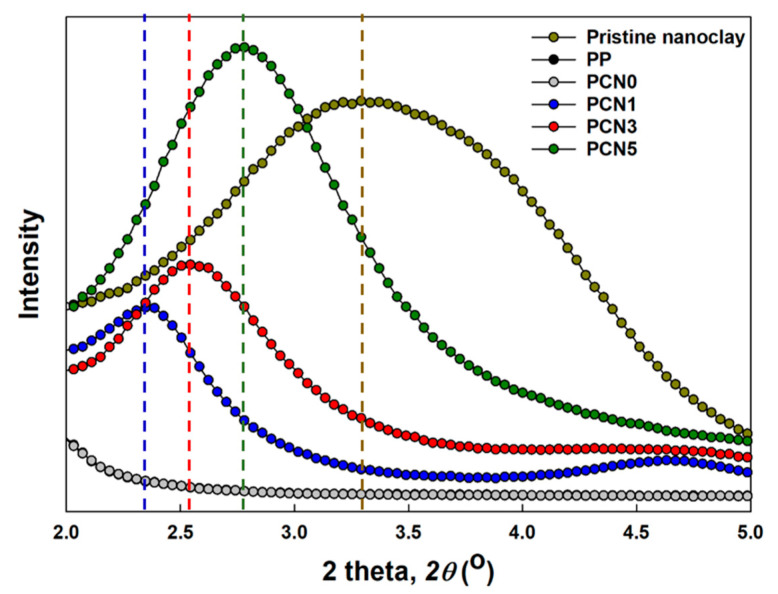
XRD spectra of pristine nanoclay, PP, PP/CNF, and PP/CNF/nanoclay nanocomposites in low 2*θ* range.

The XRD spectra presented in Figure 6 confirmed the intercalation or exfoliation characteristics in pristine nanoclay and the PP nanocomposites. Based on the XRD peaks, the 2*θ* peak, d-spacing, and full width half maximum (FWHM) values were evaluated (Table 2), while the interlayer distance (*d*_001_) was calculated using the Bragg’s equation

(1) $$n\lambda =2d_{001}\sin\theta$$

where *λ* is the wavelength, *θ* is the diffraction angle, and *d*_001_ is the interlayer distance between the nanoclays. PP and the PCN0 did not show peaks in this low 2*θ* region. Compared to pristine nanoclay, the XRD peaks inPCN1, PCN3, and PCN5, that were prepared by melt-processing, shifted at lower angles, indicating an increase in the interlayer distance due to the nanoclay intercalation or exfoliation. The nanoclay clusters in the extruder were crushed under shear stress by a mechanism called erosion and rupture, which effectively penetrates the polymer chains between the nanoclays [24]. The tendency of the d-spacing values of the PP/CNF/nanoclay nanocomposites obtained through XRD analysis was similar to that of the interlayer distance values observed from the TEM images. On average, there was a difference of 1.1-1.2 nm for each sample, because the XRD data, apart from the intercalated nanoclay, also considered the exfoliated nanoclay.

The XRD peaks confirmed that the FWHM values of the PCN1, PCN3, and PCN5 nanocomposites decreased compared to the pristine nanoclay. In addition, as the nanoclay content decreased from PCN5 to PCN1, FWHM decreased, implying that nanocomposites, i.e., more ordered nanoclay systems, are formed when the polymer chains penetrate well between nanoclays [33]. This result could also be supported by the SEM results, where it can be assumed that the nanocomposites are formed due to the promotion of the nanoclay agglomeration by the CNF with increasing nanoclay content in the nanocomposite.

### 3.4. Thermal Stability Properties

**Figure 7:**
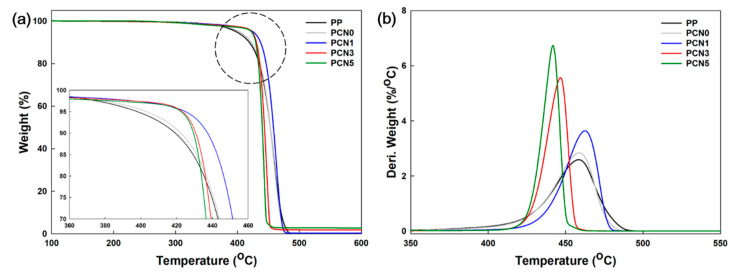
(**a**) Thermogravimetric analysis (TG) and (**b**) derivative TG (DTG) curves of PP, PP/CNF, and PP/CNF/nanoclay nanocomposites.

Figure 7 and Table 3 present the nanocomposites’ thermal properties obtained by TGA. More specifically, PCN0 did not significantly change compared to PP. However, the PCN nanocomposites exhibited different trends in contrast to PP and the PCN0 nanocomposite, because, as reported earlier, the addition of nanoclay can improve the thermal stability of composites due to the barrier effect that decelerates the diffusion of volatiles and gases [34,35].

The addition of 1 wt % nanoclay in the PC nanocomposite improved the initial decomposition temperatures, T_−5%_ and T_−10%_, by about 17 and 11 °C, respectively, compared to the nanoclay-free PCN0 nanocomposite. Similarly, PCN3 and PCN5 showed higher T_−5%_ and T_−10%_ than PCN0, but had lower values than PCN1. Based on these results, the thermal stability of PCN1, PCN3, and PCN5 was more improved than the PCN0 case by increasing the initial decomposition temperature. However, the onset temperatures (T_onset_) decreased as the nanoclay content increased, probably due to the decomposition of ammonium salt in the nanoclay and the nanoclay agglomeration as its content increased [36].

Apart from PP and the PCN0 nanocomposite that decomposed by almost 100% at 500 °C, the PCN nanocomposites exhibited different actual feed contents and residual amounts of inorganic matter after the TGA measurements. In the case of CNF, about 20% of the residue remained after decomposition at 500 °C. One of the organically treated nanoclays, cloisite 20A, consists of only 75% by weight of inorganic material, so the residue at 500 °C is 75%. Computationally, PCN0, PCN1, PCN3 and PCN5 should leave 0.2, 0.95, 2.45 and 3.95% of the residuals, respectively. Therefore, the value measured by TGA may differ from the initial amount of the added fillers.

### 3.5. Crystallization Behavior Properties

**Figure 8:**
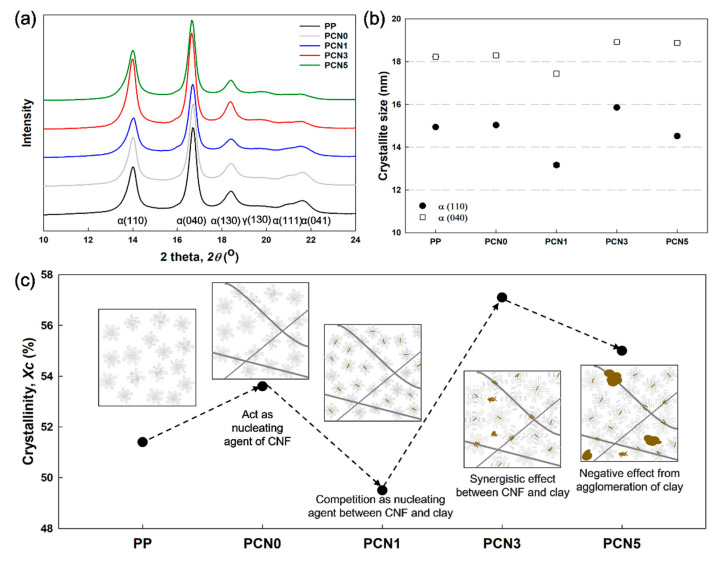
(**a**) XRD spectra of PP, PP/CNF, and PP/CNF/nanoclay nanocomposites in high 2θ range; (**b**) calculated crystallite size based on the XRD spectra; (**c**) *Xc* and predictable morphology structures.

Figure 8 shows the effect of the composition on the XRD profiles of PP and the PP/CNF and PP/CNF/nanoclay nanocomposites. The diffraction peaks detected at 2θ = 14.1°, 16.8°, 18.6°, 20°, 21.1°, and 21.8 ° were observed for all the samples and corresponded to α(110), α(040), α(130), γ(130), α(111), and α(040), respectively [37,38]. A difference in the intensities of PCN1 and PCN3 compared to other samples could be clearly observed in Figure 8a. The crystallite size and crystallinity (*Xc*) were calculated using XRD peaks, and these deviations were further confirmed. The crystallite size *L_hkl_* in the direction perpendicular to the plane was calculated by the Scherrer equation

(2) $$L_{hkl}=\left(K\lambda \right)/\left(FWHM\times \cos\theta \right)$$

where *K* is the crystal shape factor, λ is the wavelength, *FWHM* is the full width at half maximum of the diffraction peak, and *θ* is the peak position. Only the main peaks at α(110) and α(040) were calculated and the change in the crystallite size was observed. Moreover, *Xc* was calculated by the following equation

(3) $$X_{c}=\sum A_{crystal}/\left(\sum A_{crystal}+\sum A_{amorphous}\right)$$

where *A_crystal_* and *A_amorphous_* represent the fitted areas of the crystal and amorphous peaks, respectively.

The calculation results of the crystallite size and *Xc* are shown in Figure 8b,c. When 1 wt % CNF was added to the PP matrix, *Xc* increased by approximately 2.6%. Similar results have been earlier reported, where PP/CNF nanocomposites exhibited higher crystallinity than PP, while CNF served as a nucleating agent [25]. Previous studies have also demonstrated that nanoclay can also act as a nucleating agent in composites [39,40,41]. However, in this study, PCN1 exhibited a lower *Xc* than that of the PCN0 nanocomposite by about 4%. Moreover, the SEM and TEM morphological analyses confirmed that the nanoclay was uniformly distributed in PCN1. At that point, CNF and nanoclay may compete with each other as nucleating agents to produce relatively small crystals (Figure 8b,c), which may, in turn, decrease *Xc*. However, PCN3, which contained 2 wt % more nanoclay than PCN1, showed the highest *Xc* (57%) in this study. This remarkable increase in crystallinity can be attributed to two aspects. Firstly, the interaction of CNF with nanoclay (Figure 4e) may can act as a nucleating agent to produce large PP crystals, and where a nanoclay plate can be attached to the CNF in the fiber form, and can act as a starting point for the crystallization of PP. Secondly, small amounts of the fine micro-aggregated nanoclay can provide sufficient space for the generation of PP crystals without interfering with other nanoclays, which is expected to result in relatively large crystal formation and thus increase *Xc*. A similar case study has also been published, where the crystallinity was significantly increased due to the network structure between the fibrous organic nucleating agent and the nanoclay, and its synergistic effect [42]. The increase in *Xc* through the unique morphology of PCN3, in which CNF and nanoclay acted synergistically, enhanced the mechanical strength and modulus, as shown in Table 4 and Table 5. PCN5, where the maximum nanoclay agglomeration occurs, showed a slight decrease in *Xc* compared to PCN3. Thus, a low nanoclay content of 0.5 or 1 wt % in the polymer matrix favored the increase in crystallinity, whereas an excess of nanoclay content has been reported by Tarapow et al. [43] and Mohan et al. [44] to reduce the crystallinity due to the nanoclay agglomeration.

### 3.6. Mechanical Properties

The mechanical properties of the nanocomposites containing CNF and nanoclay, as obtained from the tensile and flexural tests, are summarized in Table 4. CNF acted as a reinforcing agent in the polymer matrix, increasing both the yield and the flexural strength. In addition, the mechanical properties of the polymer nanocomposites were closely correlated with *Xc*, indicating the mechanical strength increased with increasing *Xc* [45,46].

**Figure 9:**
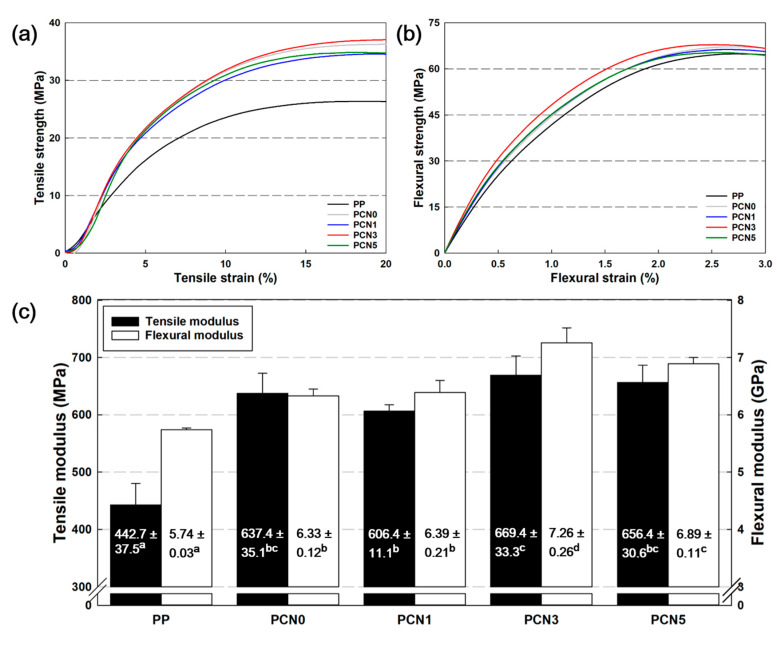
(**a**) Tensile, (**b**) flexural strain–stress (S-S) curves, and (**c**) Tensile and flexural moduli of PP, PP/CNF, and PP/CNF/nanoclay nanocomposites. (Different superscript in same column indicate that they are statistically significantly different at 95% confidence level (α = 0.05)).

In particular, based on the data of Table 4 and Figure 9, the PCN nanocomposites were closely related to *Xc*, and the difference in both the elongation at break and the two moduli (tensile and flexural) values depended on the *Xc* changes. The content of nanoclay increased from 1 to 5 wt %, showing a change in *Xc*. In PCN3, CNF and nanoclay exerted a synergistic effect as a nucleating agent, affecting the mechanical properties. As a result, in the S-S curve of Figure 9a,b, PCN3 showed a relatively higher modulus and strength than PCN0 containing CNF alone. PCN3 exhibited the best mechanical performance, and its tensile and flexural moduli were improved by 51% and 26%, respectively, compared to PP. In addition, the increased *Xc* enhanced the stiffness, which in turn decreased the elongation at break compared to PP, while no mechanical defects were detected due to the agglomeration of the nanoclay.

In a previous study, PP-g-MA contained in PP/nanoclay nanocomposites induced plasticization of the PP matrix, reducing *Xc* and tensile strength [24]. On the other hand, in the case of PP/CNF/nanoclay nanocomposites produced through plasma treatment, there was no negative effect of the reduction in mechanical (tensile and flexural) strength.

### 3.7. Barrier Properties

Table 5 shows the oxygen and water vapor permeability of PP and the PP/CNF and PP/CNF/nanoclay nanocomposites. There are several factors affecting the oxygen barrier properties, such as the polymer internal structure, crystallinity, molecular weight, and molecular entanglement. It was confirmed that the oxygen permeability of PCN3 and PCN5 was reduced compared to PCN0. Moreover, although PCN1 contained 1 wt % more nanoclay than PCN0, it was estimated that the decrease in oxygen permeability was due to the decrease in crystal size and *Xc*. In contrast to the mechanical properties, PCN5 showed higher oxygen barrier properties than PCN3, implying that the inclusion of more nanoclays increased the barrier properties by forming a more tortuous path inside the polymer.

PP had a value of 0.38 g mm/m^2^ day atm and showed excellent properties in water vapor permeability, while the PP/CNF and PP/CNF/nanoclay nanocomposites also showed similar excellent values. The surface of PP has a contact angle of about (107°) and exhibits hydrophobic properties. Thus, considering that moisture is difficult to adsorb on the hydrophobic surface, PP seems to have high water vapor barrier properties. During the formation of the PP/CNF and PP/CNF/nanoclay nanocomposites, N_2_ plasma treatment was applied to improve the affinity between CNF, nanoclay, and PP. It was also reported that the polar groups produced after plasma treatment were volatile and unstable, and they disappeared on the surface within a short time [47]. Thus, the PP/CNF and PP/CNF/nanoclay nanocomposites showed water vapor barrier properties comparable to PP.

## 4. Conclusions

In this study, PP/CNF/nanoclay nanocomposites were prepared by melt-mixing, where the filler content was fixed at 1 wt % CNF and the nanoclay was increased from 1 to 5 wt %. The morphological analyses by SEM and TEM verified that the CNF induced the agglomeration of nanoclay as the content of nanoclay increased, while the relatively decreasing tendency of the thermal stability, as observed by TGA analysis, supported these results. The nanocomposites’ morphology changed depending on the nanoclay content and, based on the results of various analyses, the nanoclay content of 3 wt % in PCN3 was demonstrated as the optimum content. PCN3 showed the highest *Xc* and crystal size compared to PP, PCN1, and PCN5, because CNF and nanoclay acted synergistically as a nucleating agent. The increased *Xc* of PCN3 improved the mechanical properties, i.e., the tensile and flexural moduli increased by about 51 and 26%, respectively, compared to PP. The oxygen barrier property was also improved in comparison to the nanoclay-free PP/CNF nanocomposite when a nanoclay amount of ≥3 wt % was further added to the PP matrix. This resembled the synergistic effect of CNF and nanoclay in hybrid systems and revealed that the combination of CNF and nanoclay can be a promising filler to enhance the composite performance in food packaging applications.

## Figures and Tables

**Table 1:** Composition of the polypropylene (PP)/cellulose nanofiber (CNF) and PP/CNF/nanoclay nanocomposites.

Sample Code	Suspensions (CNF, g/Nanoclay, g)	Final Composition of Nanocomposites (PP, wt %/CNF, wt %/Nanoclay wt %)
PCN0	CNF suspension (0.4/0)	99/1/0
PCN1	CNF/nanoclay suspension (0.4/0.4)	98/1/1
PCN3	CNF/nanoclay suspension (0.4/1.2)	96/1/3
PCN5	CNF/nanoclay suspension (0.4/2.0)	94/1/5

**Table 2:** Nanoclay interlayer characteristics by XRD measurements.

Sample Code	2*θ* Peak (°)	d-Spacing of Nanoclay, *d*_001_ (nm)	FWHM
Pristine nanoclay	3.27	2.70	2.23
PCN1	2.35	3.76	0.83
PCN3	2.54	3.47	0.97
PCN5	2.78	3.18	1.21

**Table 3:** Thermal properties of PP, PP/CNF, and PP/CNF/nanoclay nanocomposites.

Sample Code	T_−5%_ (°C)	T_−10%_ (°C)	T_onset_ (°C)	Residue Amounts (%)
PP	401.5 ± 5.1 ^a^	420.9 ± 2.6 ^a^	434.6 ± 1.1 ^ab^	0.1 ± 0.0 ^a^
PCN0	408.1 ± 5.2 ^a^	425.3 ± 3.0 ^ab^	437.2 ± 1.4 ^b^	0.1 ± 0.1 ^a^
PCN1	425.4 ± 0.9 ^b^	436.5 ± 0.9 ^c^	443.7 ± 1.2 ^c^	0.9 ± 0.1 ^b^
PCN3	422.1 ± 4.3 ^b^	430.1 ± 2.2 ^b^	435.1 ± 0.9 ^ab^	2.5 ± 0.2 ^c^
PCN5	422.2 ± 0.0 ^b^	429.4 ± 0.0 ^b^	433.7 ± 0.0 ^a^	3.9 ± 0.1 ^d^

* Different superscript in same column indicate that they are statistically significantly different at 95% confidence level (α = 0.05).

**Table 4:** Mechanical properties of PP, PP/CNF, and PP/CNF/nanoclay nanocomposites.

Sample Code	Tensile Stress at Yield Stress (MPa)	Elongation at Break (%)	Flexural Strength (MPa)	Flexural Strain at Flexural Strength (%)
PP	26.69 ± 0.77 ^a^	935.24 ± 38.58 ^d^	64.58 ± 0.42 ^a^	2.66 ± 0.08 ^c^
PCN0	36.44 ± 0.18 ^c^	416.56 ± 82.83 ^b^	66.68 ± 0.69 ^bc^	2.58 ± 0.02 ^bc^
PCN1	34.73 ± 0.56 ^b^	605.95 ± 13.84 ^c^	65.65 ± 0.90 ^ab^	2.56 ± 0.05 ^ab^
PCN3	36.61 ± 0.55 ^c^	289.79 ± 65.85 ^a^	67.44 ± 0.55 ^c^	2.47 ± 0.05 ^a^
PCN5	34.62 ± 0.63 ^b^	499.06 ± 66.34 ^bc^	65.53 ± 0.39 ^ab^	2.47 ± 0.05 ^ab^

* Different superscript in same column indicate that they are statistically significantly different at 95% confidence level (α = 0.05).

**Table 5:** Oxygen and water vapor permeability of PP and the PP/CNF and PP/CNF/nanoclay nanocomposites.

Sample Code	Oxygen Permeability (cc-mm/m^2^-Day-atm)	Water Vapor Permeability (g-mm/m^2^-Day-atm)
PP	132.66 ± 0.79 ^c^	0.38 ± 0.02 ^a^
PCN0	107.02 ± 6.78 ^b^	0.37 ± 0.01 ^a^
PCN1	106.71 ± 2.94 ^b^	0.39 ± 0.05 ^a^
PCN3	95.77 ± 0.97 ^a^	0.39 ± 0.03 ^a^
PCN5	92.95 ± 1.58 ^a^	0.37 ± 0.02 ^a^

* Different superscript in same column indicate that there are statistically significantly different at 95% confidence level (α = 0.05).
